# Latex vascular injection as method for enhanced neurosurgical training and skills

**DOI:** 10.3389/fsurg.2024.1366190

**Published:** 2024-02-23

**Authors:** Julio C. Pérez-Cruz, Mario A. Macías-Duvignau, Gervith Reyes-Soto, Oscar O. Gasca-González, Matias Baldoncini, Franklin Miranda-Solís, Luis Delgado-Reyes, Carlos Ovalles, Carlos Catillo-Rangel, Evgeniy Goncharov, Renat Nurmukhametov, Michael T. Lawton, Nicola Montemurro, Manuel De Jesus Encarnacion Ramirez

**Affiliations:** ^1^ Laboratorio de Técnicas Anatómicas y Material Didactico, Escuela Superior de Medicina, Instituto Politécnico Nacional, Mexico City, Mexico; ^2^ Departamento de Anatomía, Facultad de Medicina, Universidad Nacional Autónoma de México, Mexico City, Mexico; ^3^ Department of Head and Neck, Unidad de Neurociencias, Instituto Nacional de Cancerología, Mexico City, Mexico; ^4^ Departamento de Anatomía, Instituto de Seguridad y Servicios Sociales de los Trabajadores del Estado, Mexico City, Mexico; ^5^ Laboratory of Microsurgical Neuroanatomy, School of Medicine, University of Buenos Aires, Buenos Aires, Argentina; ^6^ Laboratorio de Neuroanatomía, Centro de Investigación de Anatomía y Fisiología Alto Andina, Universidad Andina del Cusco, Cusco, Peru; ^7^ Department of Neurosurgery, General Hospital, Durango, Mexico; ^8^ Department of Neurosurgery, Servicio of the 1ro de Octubre Hospital of the Instituto de Seguridad y Servicios Sociales de los Trabajadores del Estado, Mexico City, Mexico; ^9^ Traumatology and Orthopedics Center, Central Clinical Hospital of the Russian Academy of Sciences, Moscow, Russia; ^10^ Neurological Surgery, Peoples Friendship University of Russia, Moscow, Russia; ^11^ Department of Neurosurgery, St. Joseph’s Hospital and Medical Center, Barrow Neurological Institute, Phoenix, AZ, United States; ^12^ Department of Neurosurgery, Azienda Ospedaliero Universitaria Pisana (AOUP), Pisa, Italy

**Keywords:** latex vascular injection, microsurgery, neurosurgery, training, specimen, surgical technique

## Abstract

**Background:**

Tridimensional medical knowledge of human anatomy is a key step in the undergraduate and postgraduate medical education, especially in surgical fields. Training simulation before real surgical procedures is necessary to develop clinical competences and to minimize surgical complications.

**Methods:**

Latex injection of vascular system in brain and in head-neck segment is made after washing out of the vascular system and fixation of the specimen before and after latex injection.

**Results:**

Using this latex injection technique, the vascular system of 90% of brains and 80% of head-neck segments are well-perfused. Latex-injected vessels maintain real appearance compared to silicone, and more flexible vessels compared to resins. Besides, latex makes possible a better perfusion of small vessels.

**Conclusions:**

Latex vascular injection technique of the brain and head-neck segment is a simulation model for neurosurgical training based on real experiencing to improve surgical skills and surgical results.

## Introduction

Tridimensional knowledge of human anatomy is a key step in undergraduate and postgraduate medical education. This knowledge is even more important in surgical fields, where the use of corpses is a great source to develop real tridimensional anatomy knowledge, being last essential in training surgical approaches. Both issues are important in the education of future and current specialists. In neurosurgical practice, the knowledge of vascular system and its muscle, bony, meningeal, cisternal, and ventricular relationships related to neurosurgical approaches are vital (1). The experience of training simulation before real surgical procedures is necessary to enhance clinical competences at undergraduate and postgraduate levels (2, 3). First vascular anatomy studies date back to XVII century where authors as Humphrey Ridley, Thomas Willis, and Richard Lower made selective injections of the arterial system (4). Meanwhile, Leonardo da Vinci injected and made molds from the cerebral ventricles (5). Historically, many materials have been used to the injection of the cerebral vascular system. These include mercury, spermaceti, tallow, latex, epoxy resins, and silicone, last three definitely the most used (4–6). Cerebral vascular system injection is very helpful for the study of microsurgical vascular cerebral anatomy, as well as the muscle, bony, meningeal, cisternal, and ventricular relationships for viewing, through surgical microscope, the collateral and perforating branches with precision (7–11).

Recent advancements in neurosurgical training have focused on developing alternative methods that can supplement or even replace traditional cadaver-based learning (4). One such innovation is the use of synthetic or semi-synthetic models that mimic the physical properties of human tissues (12, 13). These models can be engineered to replicate the complex anatomy of the human brain and its vascular structures, offering a more accessible and sustainable option for training purposes. Additionally, advancements in 3D printing technology have opened new avenues for creating detailed anatomical replicas (14, 15). These 3D-printed models can be customized to simulate specific pathological conditions or unique anatomical variations, thereby enhancing the depth and breadth of surgical training (16, 17). While technological advancements and alternative methods in neurosurgical training are making significant strides, the cadaveric method remains arguably the most efficient and effective approach for developing a deep and practical understanding of human anatomy, especially in the context of neurosurgery (18, 19).

The cadaveric method allows for a hands-on approach to learning, which is essential in a field as intricate as neurosurgery (20–22). Two processes can be used to obtain biological cadaveric simulators for neurosurgical training: partial necropsy and injection of the head-neck segment.

In this paper, we describe our latex vascular injection technique of the brain and the head-neck segment as simulators for neurosurgical training, a very helpful tool in neurosurgical formation that makes possible a real scenario with soft vessels for a correct dissection and even can simulate bleeding. The primary objective of this paper is to focus on the methodologies employed for latex vascular injection in brain and head-neck segments, whereas the secondary objectives are the outcome of these techniques and their implications for neurosurgical training. The steps involved in the process are discussed, from extraction and washing to fixation, latex injection, storage and finally dissection of the specimens.

## Materials and methods

### Type of study

This research is characterized as a descriptive, experimental study focused on the development and refinement of neurosurgical training techniques. Our primary objective is to optimize the realism and educational value of neurosurgical simulations through the innovative use of the latex vascular injection method. We meticulously document the anatomical features and variations observed in the cadaveric specimens during and after the application of our methods. This includes detailed descriptions of the vascular structures and other relevant neuroanatomical features, providing valuable insights for educational purposes. The cadaveric specimens used in this study are selected based on specific criteria to ensure consistency and relevance to neurosurgical training. Each specimen undergoes a series of preparatory steps, including perfusion, fixation and latex injection, which are crucial for the success of the experimental process. For this study, we included human cadaveric specimens with no restrictions on age or gender, specimens from individuals who did not suffer from neurodegenerative diseases or head trauma were included to ensure the integrity of the cerebral and neck vascular systems, specimens obtained within a specified postmortem interval, preferably not exceeding 24 h to minimize tissue degradation, specimens that have not undergone any prior preservation processes or dissections to ensure the integrity of anatomical structures. Exclusion Criteria are specimens with a known history of neurological disorders, head injuries or surgeries that could alter the standard anatomy, specimens showing signs of advanced decomposition or significant tissue degradation, specimens previously exposed to chemicals or preservation agents that could interfere with the latex injection process or the subsequent dissection. A total of 60 specimens (25 for brain injection and 25 for head-neck injection) were included in this study.

### Brain injection technique

Brain injection is a 7-step method as following: (1) extraction; (2) wash out; (3) fixation by perfusion-immersion; (4) latex injection; (5) fixation by immersion; (6) preservation; (7) microsurgical dissection.

#### Extraction

It is very important to avoid cerebral vascular system injury while extraction from the cranial cavity. Cranial nerves, arteries, veins, and venous sinuses must be cut near to the base of the skull and fair from the brain. We recommend non-traction during exposition of structures before cutting. Before cutting following structures must be identified to void their damage: cerebral portion of the internal carotid artery, internal auditory artery, fourth portion of the vertebral artery (V4), superficial middle cerebral vein, superior petrous sinus, major petrosal vein, and transverse sinus. Non-traction of the tentorium cerebelli is necessary to maintain rectus sinus, great cerebral vein (great vein of Galen), and pineal gland at their original position. Depending on the experience of the anatomist and the condition of the specimen, this step can take from 30 min to 1 h.

#### Washing

**Figure 1:**
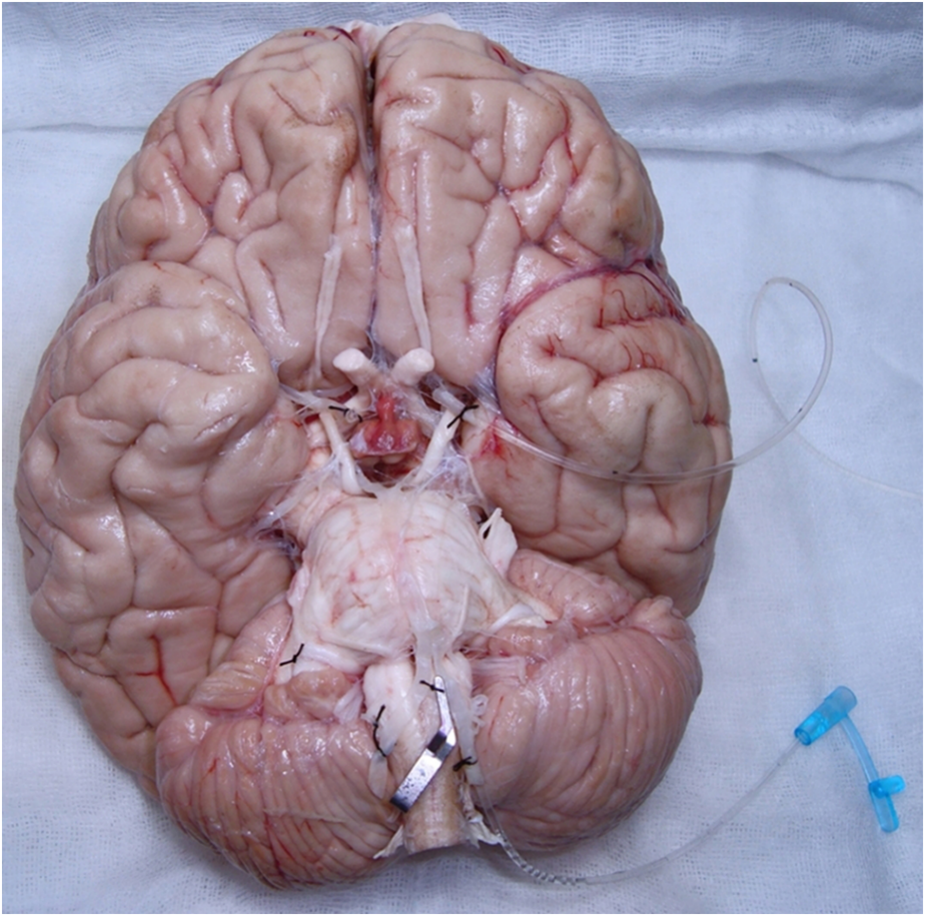
Catheterization of the vertebral and internal carotid arteries for cerebral artery system washing out.

After successfully obtaining the brain, the next critical step is the catheterization of one vertebral artery, which is performed using a 5 French (Fr) catheter, similarly applied to one internal carotid artery. Concurrently, the contralateral vertebral and internal carotid arteries are securely closed. For this closure, we recommend the use of black silk, which has proven to be effective for this purpose. Following this, the arterial system undergoes a thorough washing process using a saline solution. This step is essential to remove any remaining blood and clots from the arteries. The washing is conducted manually with a syringe, injecting the saline solution continuously and generously (Figure 1). Care is taken to ensure that the pressure is not excessive, as this could potentially lead to the rupture of smaller vessels, which are particularly vulnerable to damage. During the saline perfusion of the arterial system, it is crucial to identify and promptly seal any leakage sites. These sites typically correspond to the arteries that were cut during the extraction process. Special attention is required for the internal auditory artery, which is often a site of significant leakage. The closure of these leakage points is achieved through various methods, including ligation with black silk, the use of vascular clips, or the application of cyanoacrylate. Each of these techniques has its own advantages and is selected based on the specific requirements of the leakage site. This process of catheterization, closure, and washing is meticulously carried out to ensure the integrity of the vascular system and the success of subsequent steps in the brain preparation for neurosurgical training simulations. The washing process typically takes about 30 min to 1 h, including the time to secure catheters and perform the washing.

#### Fixation by perfusion-immersion

The fixation process is a crucial step in preparing the brain for neurosurgical training simulations and consists of three distinct stages: perfusion, immersion, and perfusion during immersion. Each stage is meticulously designed to ensure optimal preservation of the brain's anatomical integrity. The initial perfusion step in brain dissection techniques, particularly when using formaldehyde or similar fixatives, is a critical procedure that underpins the effectiveness of the entire preservation and dissection process. This step is not merely a preparatory action but a fundamental technique that significantly enhances the anatomical and functional value of the brain specimen for neurosurgical training and research. The reasons for this are multifaceted and deeply rooted in the biological and chemical properties of brain tissue and the fixatives used. This is crucial for preserving the brain's vascular architecture, which is a critical component of neurosurgical training. Moreover, the perfusion of fixatives ensures a uniform distribution of these chemicals throughout the brain tissue.

Perfusion: Initially, with the vertebral and internal carotid arteries already catheterized, 15 milliliters (ml) of pure formaldehyde are perfused through these vessels. This perfusion is carried out simultaneously for a duration of 5 min. The purpose of this step is to ensure that the formaldehyde, a potent fixative, permeates the vascular system of the brain, thereby initiating the fixation process at a vascular level. Initiating the fixation process through perfusion also plays a critical role in halting autolysis and decomposition. By introducing formaldehyde or a similar fixative into the brain's vasculature, these enzymes are denatured, effectively halting the breakdown process. This preservation of cellular and tissue integrity is essential for maintaining the morphological details of the specimen, which are essential for accurate anatomical training and surgical practice.

Immersion: Following the perfusion, the brain is then placed in a container filled with 3 L of a 10% formaldehyde solution. To prevent the brain from touching the bottom of the container, which could potentially lead to deformations or damage to its anatomy, gauze is strategically placed to keep the brain suspended. This immersion in formaldehyde solution lasts for 15 min, allowing the fixative to penetrate the brain tissue more deeply and uniformly.

Perfusion during immersion: In the final stage, an additional 1 L of 10% formaldehyde solution is perfused through the internal carotid artery while the vertebral artery remains open. This setup allows the formaldehyde to have an exit route, ensuring that the solution flows throughout the brain's vascular system. This perfusion is conducted over 30 min using an infusion system, which helps maintain a consistent flow rate. During this stage, the brain benefits from a dual fixation process: it is fixed by immersion in the formaldehyde bath and by the continuous perfusion of the solution through its vascular system. This three-step fixation method is carefully arranged to preserve the brain's structural integrity, allowing for a more realistic and detailed study during neurosurgical training. The combined effects of perfusion and immersion ensure that the brain is adequately prepared for subsequent procedures, such as latex injection and microsurgical dissection, while maintaining the fidelity of its intricate anatomical features.

#### Latex injection and fixation by immersion

Once the brain has been fixed, it must be removed from solution to start latex injection. White latex (Poliformas plásticas®) is mixed 1:1 with carmine 319 acrylic paint (Politec®), and 15 ml of this mix is perfused through vertebral artery, and 20 ml through internal carotid artery, once at a time. If there is any leakage of latex it must be washed immediately with running water to avoid latex impregnation of arachnoid or pia mater. Latex is easily cleared by water if latex is fresh. Never wash latex with hot water as latex hardens with heat and get fastened to arachnoid. At the end of latex injection, catheters are withdrawn, immediately internal carotid and vertebral arteries are ligated using black silk. After latex injection, brain most be submerged once again in 10% formaldehyde solution. After 24 h, solution is change by a new 10% formaldehyde solution in which brain will be for 2 months before dissection. Depending on the complexity of the vascular system and the need to manage leaks, this can take 1–2 h.

#### Preservation and subsubsection

After 2 months in 10% formaldehyde solution, brain washing for 24 h using running water is required to eliminate formaldehyde. Then, brain is preserved onwards in a 60% isopropyl alcohol solution. For vascular system dissection we recommend the use of a microsurgical microscope, Rothon's dissectors, fine scissors and microsurgery tweezers. In this step, we make a strong recommendation for photographical documentation before, during, and after dissection for posterior processes and anatomy analysis. The time required is 24 h for washing, followed by indefinite preservation until dissection.

### Head-neck segment injection technique

Head-neck segment injection is also a 7-step technique, where the last 5 steps are similar to the brain injection. Minor differences in those steps are mentioned: (1) selection of the body and vascular dissection in cervical region; (2) washing of vascular system of head-neck segment; (3) fixation by perfusion; (4) latex injection; (5) fixation by immersion; (6) preservation; (7) microsurgical dissection.

#### Selection of the body and vascular dissection in cervical region

The human body cause of death must be not a neurologic-related, and with a post-mortem period less than 24 h. A 5 cm-incision is made on lower third and anterior edge of the sternocleidomastoid muscle for vascular dissection of the common carotid artery, vertebral artery, internal jugular vein, and external jugular vein. Then, an 8 Fr catheter that is fixed with 2-0 black silk is used to catheterize mentioned vessels.

#### Washing of vascular system of head-neck segment

This is a manual washing using a syringe and it is started in right common carotid artery with either 180 ml of water or saline solution for blood elimination. Water will exit from left common carotid and vertebral arteries. Then, 180 ml of water are perfused through left common carotid artery, then 60 ml through every vertebral artery, once at a time. This washing is made slowly and continuously without excess of pressure, the last could lead to the rupture of small-size vessels. After arterial washing, vein washing is performed. For this, 240 ml of water are perfused through right internal jugular vein while water exits from left internal jugular vein. Then, 240 ml of water are perfused through left internal jugular vein while water exits from right internal jugular vein. The same process is made in external jugular veins, right first and then left but only with 120 ml of water in each one. The washing process typically takes about 30 min to 1 h, including the time to secure catheters and perform the washing, as we said above.

#### Fixation by perfusion and latex injection

After washing of the vascular system of head-neck segment, a formula for preservation of the Medicine Superior School of the National Polytechnic Institute from Mexico (Escuela Superior de Medicina del Instituto Politécnico Nacional, ESM-IPN) is perfused through right common carotid artery by a Portiboy Mark IV® perfusion bomb, this solution exits from contralateral common carotid artery. White latex (Poliformas plásticas®) is mixed 1:1 with either carmine 319 acrylic paint (Politec®) for arteries or ultramar blue 315 acrylic paints (Politec®) for veins. For arteries, 60 ml of this mix is perfused through vertebral artery; and 120 ml through carotid artery system, for adequate perfusion of cerebral vessels it is important to see conjunctival and sublingual mucosal vessels perfusion. If there is any abundant leakage of latex, vessels must be closure by either 2-0 black silk, or hemostatic tweezers, or vascular clips.

#### Fixation by immersion, preservation and microsurgical dissection

Once latex injection is completed, body is placed in a container full of ESM-IPN solution for two months. After the two months of fixation by immersion, washing of head-neck segment is made for 24 h with running water. Then, segment is preserved in 60% isopropyl alcohol solution. Microsurgical instrumental is a helpful tool for the segment dissection. We recommend supracerebellar-supratentorial approach by suboccipital craniotomy, then cut Dura matter, open and dissect cisterna magna to identify neural, cisternal, arterial, venous, and meningeal elements.

### Ethical considerations

The study was conducted in accordance with the Declaration of Helsinki and approved by the Ethics Committee of the Department of Head and Neck, Unidad de Neurociencias, Instituto Nacional de Cancerología, Mexico City, Mexico. The study adheres to strict ethical guidelines regarding the use of cadaveric specimens, with all necessary permissions and consents obtained in accordance with institutional and legal requirements.

## Results

**Figure 2:**
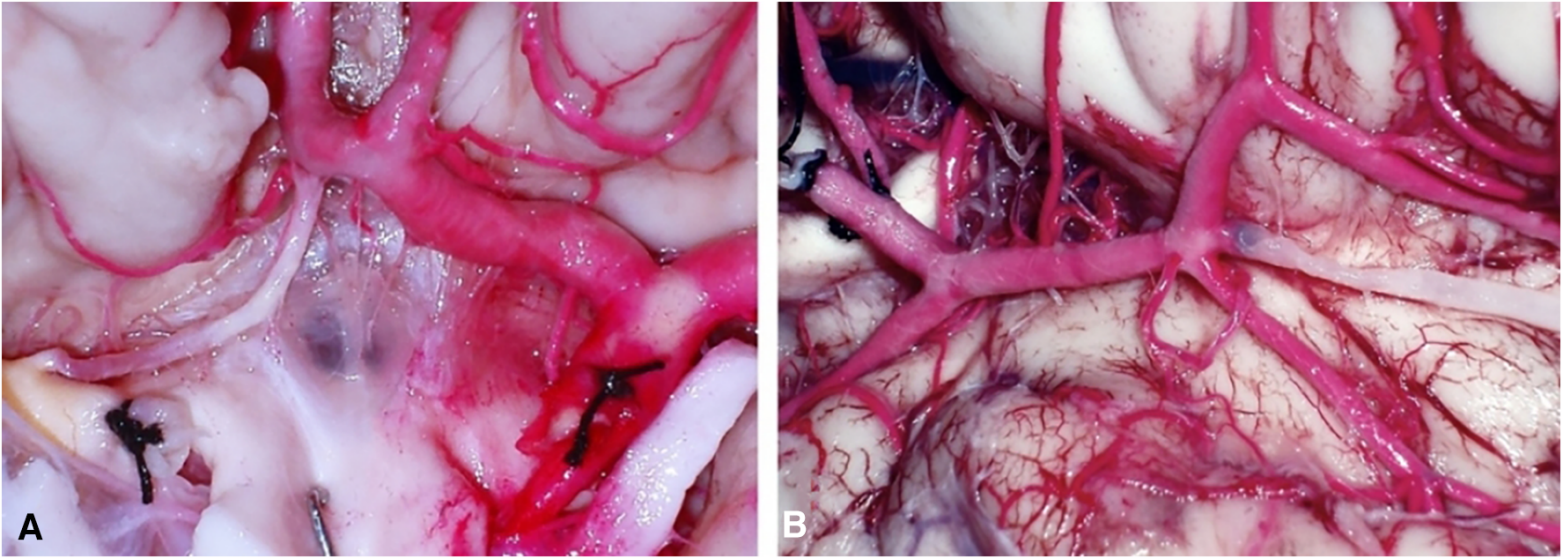
(**A**) Hypoplasia of right A1 (rA1) and atherosclerotic plaques at right internal carotid artery (rICA). (**B**) Dissection of Sylvian fissure region: a clot was found in a collateral branch of the first segment of middle cerebral artery (M1).

Using these techniques, 90% of the brains are well perfused. Both supra and infratentorial systems, as vertebrobasilar system, Willis polygon, M1, M2, and M3 segments of middle cerebral artery, A1–A4 segments of anterior cerebral artery, posterior cerebral artery, and anterior choroidal artery are well-perfused. Besides an optimal technique, 10% of the brains have a non-well latex superfusion due to anatomical variations as artery hypoplasia, atherosclerotic plaques in arteries or persistence of clots in the vascular system (Figure 2). For head-neck segment injection, we found that 20% of cases had extravasation of latex due to cutting of small vessels in frontal-parietal-temporal region. The rest cases had an adequately latex superfusion of intracranial and extracranial vascular system. Using this technique, all segments had a well latex superfusion of infratentorial vascular system.

The latex vascular injection method presents an innovative approach to neurosurgical training, offering high realism and educational value, especially for vascular anatomy and surgical techniques. However, it requires careful preparation and handling to maintain the quality and longevity of the specimens. On the other hand, the head-neck injection method provides a comprehensive anatomical model that is beneficial for a broader range of neurosurgical training objectives, though it may involve more complex preparation processes and potentially higher costs due to the need for skilled personnel (Table 1). Both methods have their unique advantages and limitations, and the choice between them should be based on the specific educational goals, available resources, and logistical considerations of the training program.

**Table 1:** Pros and cons between the latex vascular injection method and the head-neck injection method.

Criterion	Latex vascular injection method	Head-neck injection method
Realism	Pros: High realism in vascular perfusion, allowing better visualization of small vessels. Latex flexibility simulates vessel elasticity.	Pros: Offers realistic anatomical context by preserving the entire head-neck segment, including neural, arterial, venous, and meningeal elements.
	Cons: Increased tissue stiffness due to formaldehyde fixation may reduce overall realism in tissue texture.	Cons: May offer less flexibility in vessels compared to latex injection, potentially reducing realism in vascular manipulation.
Preparation complexity	Pros: Standardized method with detailed steps for specimen preparation, from fixation to latex injection.	Pros: Comprehensive approach that includes vascular system washing, fixation, and injection, applicable to entire head-neck anatomy.
	Cons: Requires meticulous handling to prevent latex leakage and ensure proper perfusion, which may increase the complexity of preparation.	Cons: Complex dissection and preparation due to the inclusion of both arterial and venous systems, requiring careful handling.
Durability	Pros: Latex injection provides durable vascular models for long-term educational use. Formaldehyde fixation enhances tissue preservation.	Pros: Robust preservation techniques ensure long-term durability of specimens for repeated educational use.
	Cons: Latex may degrade over time if not properly stored, potentially affecting the longevity of the specimen.	Cons: Specimens may require maintenance and proper storage to prevent degradation, especially in regions with high humidity.
Cost-effectiveness	Pros: Latex and formaldehyde are relatively inexpensive materials, making this method cost-effective for creating multiple specimens.	Pros: Utilizes readily available materials and techniques, making it a cost-effective option for comprehensive neurosurgical training.
	Cons: The labor-intensive preparation process may increase overall costs due to the need for skilled personnel.	Cons: Requires significant time and expertise for preparation, potentially increasing labor costs.
Educational value	Pros: Enhances understanding of microvascular anatomy and surgical techniques through detailed visualization of the vascular system.	Pros: Offers a holistic view of the head-neck anatomy, beneficial for training in a wide range of neurosurgical procedures.
	Cons: Limited to vascular anatomy and may not fully address the complexity of neurosurgical procedures involving other anatomical structures.	Cons: While comprehensive, the complexity of the specimen may overwhelm beginners, potentially diluting focus on specific learning objectives.

## Discussion

For anatomical and physiological investigations and teaching issues, the use of vascular system injection begun after William Harvey developed blood circulation doctrine at XVI century (22). Cole made exhaustive research about developing of this technique in The History of Anatomical Injections (5). Some of the pioneers of vascular injection technique include Frideric Ruysch (23), successor of Isaac Newton in the Academy of Science in France, distinguished for his ability in arterial and venous injections as in corrosions (5, 22). On the other hand, J. Swammerdam (1637–1680) was the first anatomist that used the injection of solidifying materials; Sir Charles Bell (1774–1842) used mercury for lymphatic vessels injection (5, 9).

**Figure 3:**
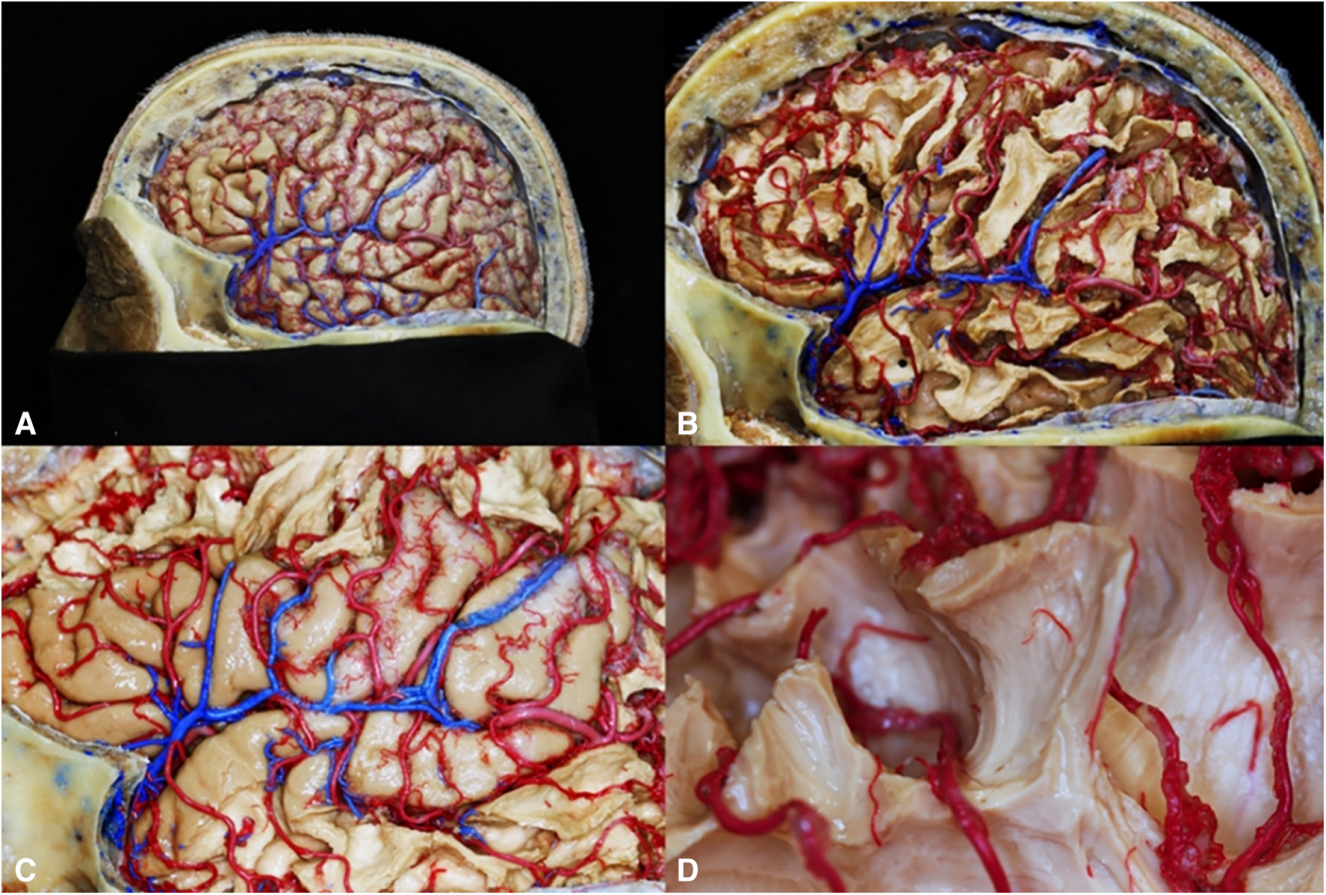
Macro and microvasculature of the cerebral white matter. (**A**) Cortical artery; (**B**) White matter superficial vascularization; (**C**) Venous drainage of the opercular region; (**D**) Microvasculature of short association fibers.

**Figure 4:**
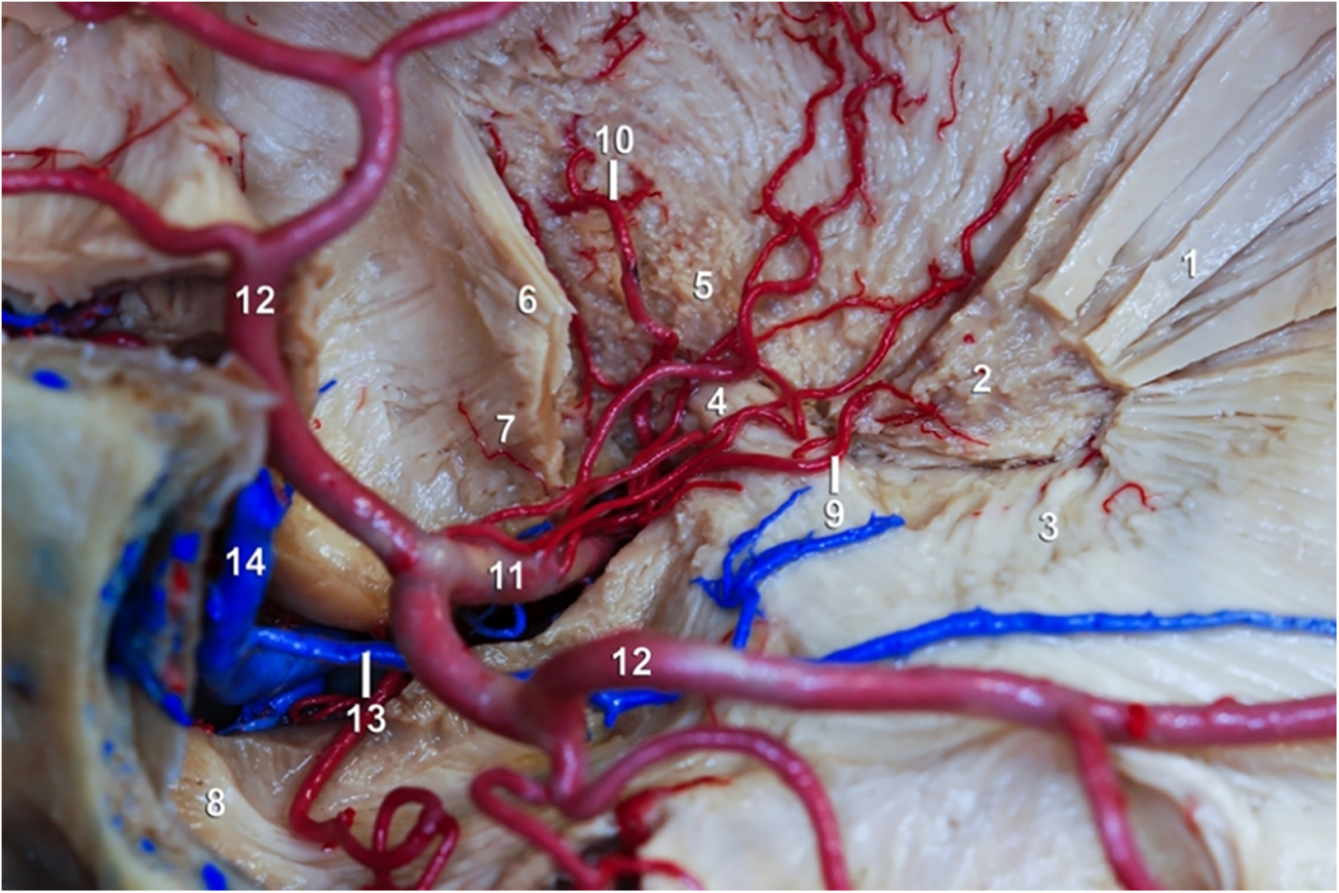
Lenticulostriate artery and white matter. (1) External capsule; (2) Putamen; (3) Meyer's handle; (4) Anterior commissure; (5) Pallid globe; (6) Inferior occipitofrontal fascicle; (7) Uniform fascicle; (8) Temporal pole; (9) Lateral lenticulostriate artery; (10) Heubner's recurrent artery; (11) M1 segment of the ACM; (12) Temporary branch of the ACM; (13) Deep Silvian vein; (14) Superficial Silvian vein.

**Figure 5:**
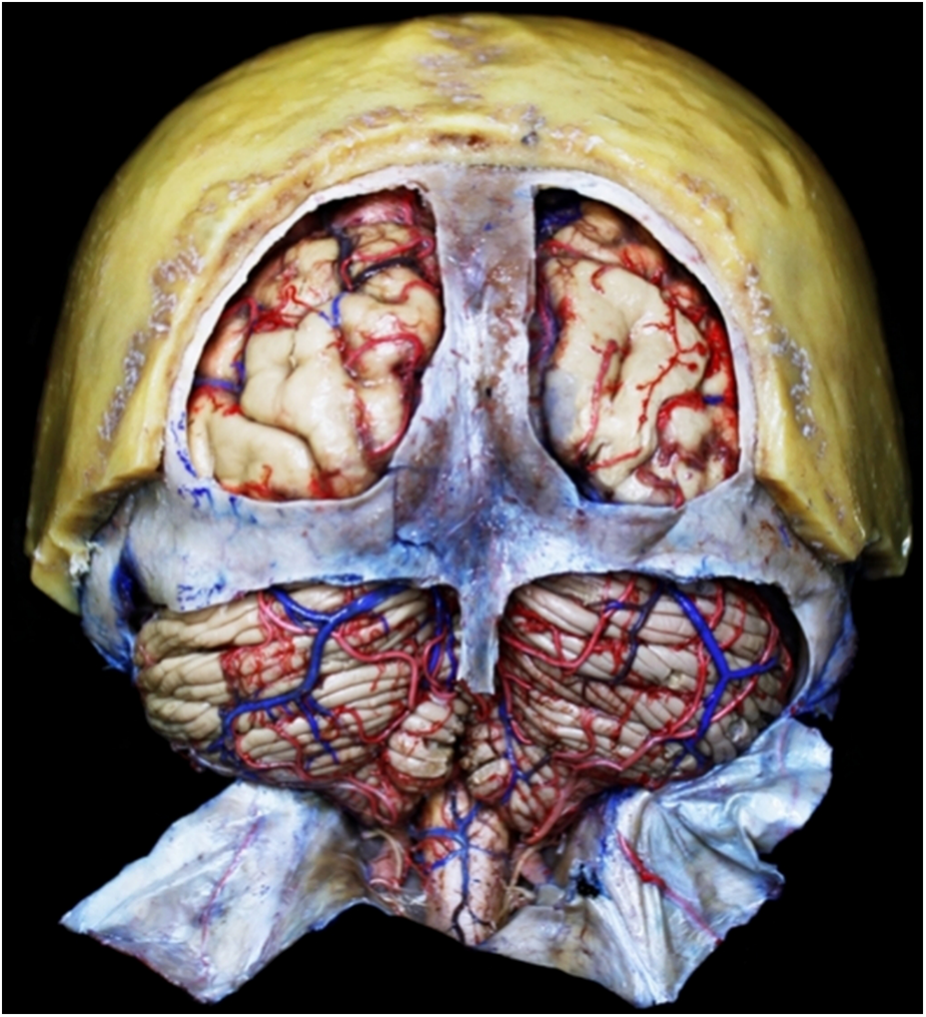
Supracerebellar-transtentorial approach.

There are a variety of substances used for vascular injection depending on either necessities or objectives. Substances that solidify by cooling as jelly or wax can be used too. Another kind of substances are those that do not alter their state of matter, as mercury; also, there are substances that solidify by polymerization, as resins, silicone, or latex; there are radiopaque substances as barium and iodine which can be used for vascular injection (23). Once brain has been injected with latex, it can be used for either developing of investigation protocols about microsurgical anatomy from the macro and microvasculature of the cerebral white matter (Figure 3), lenticulostriate artery (Figure 4) or training of surgical approaches (Figure 5). Authors do not recommend veins-injection in the brain once it has been extracted due to poor results because cortical veins are often injured during extraction procedure. For vein-injection of the brain, the preferred technique is head-neck segment injection (24). We recommend latex because it is a substance that can be perfused easily into vessels due to its low viscosity making possible injection of small vessels. Once latex has solidified, it is flexible and vessels can be manipulated during dissection without breaking off (25). Latex advantages include low cost, high availability, and nontoxicity. Also, it is soluble in water, so if there is any leakage, it can be washed during injection for total elimination (26). Vascular washing is the most important step in latex vascular injection; a correct washing in the postmortem period makes possible the vascular injection weeks, even months, after body preservation.

One of the most important limitations of human body use as a surgical simulator is the hardness of tissues after preservation formula application. In development of surgical-techniques teaching during XX century, references about limitation of cadaveric simulator use are frequent because of hard viscera due to either formalin use, putrefaction or tissue fragility; besides other factors which do not simulate real conditions, as the absence of bleeding or inflammation after tissue manipulation (27). In 1992, Thiel published a body preservation method useful in surgical training. This method allows flexibility tissue, preservation of muscle's, viscera's, and vessel's color with antimicrobial properties (28). However, this formula does not preserve brain; recently, a modification of Thiel's technique was published in which a 10% formaldehyde solution is perfused through a trepanation while body is preserved with Thiel's formula by immersion and vascular perfusion (29). Thiel's method can be used in surgical training of laparoscopy, arthroscopy, abdominal, thoracic, and pelvic procedures (30). We consider that Thiel's technique modification for brain preservation is not better than other techniques for neurosurgical training because it is necessary to use formaldehyde solution. The use of ESM-IPN formula for body preservation allows brain's tissue flexibility for retraction without tissue damage; it facilitates dissection in a supracerebellar-transtentorial approach to anterior temporo-mesial region (31). In 2002, Emad Aboud published a new neurosurgical training model to simulate cerebral vascular system in a real setting including bleeding and pulse. Using Aboud's model, neurosurgical approaches can be made with realistic anatomy of skull and brain, meanwhile it is possible to make anastomosis or place artificial aneurysms for clipping (32). Latex vascular injection makes possible the training of microsurgical procedures as termino-terminal anastomosis, with exit of latex simulating bleeding if procedure is making within 15 days after latex perfusion.

**Figure 6:**
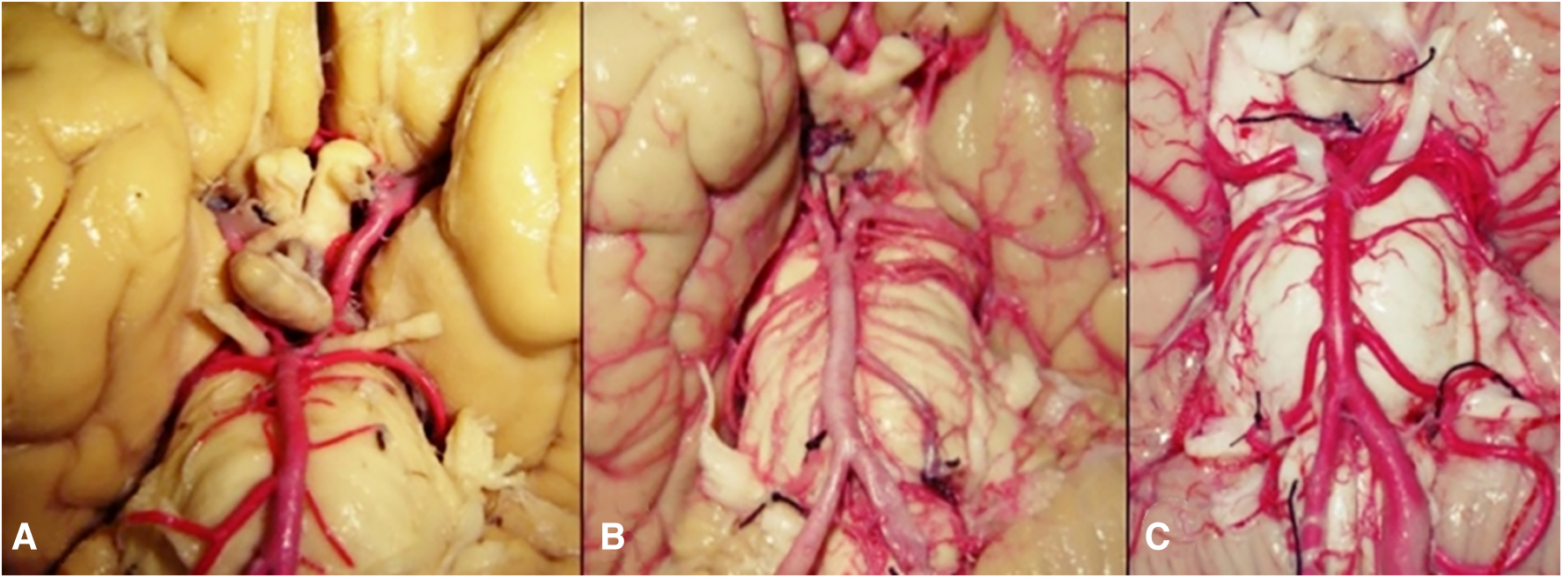
Comparison of (**A**) silicone-, (**B**) epoxy resin- and (**C**) latex- injected vessels.

Use of these simulators and procedure repetition helps to quicken the teaching-learning process in surgical approaches improving surgical time, precision, anatomic knowledge and safety of neurosurgical residents and even neurosurgeons in the surgical procedure (33, 34). Latex injection can be used also for investigations. Besides latex, other substances have been used for vascular injection of the head and brain as silicone and epoxy resin; these materials have been used for vascular and anatomical studies (35). In our laboratory we have used latex, silicone and epoxy resins for vascular injection of the brain and head-neck segment (Figure 6). Each material has different advantages and disadvantages: with silicone vessels acquires a bigger size that is very beautiful and provides an excellent visual effect for publications because while dries, in addition silicone does not collapse (36); on the other hand, silicone disadvantages include its higher viscosity that makes its perfusion more difficult and does not perfuse small vessels and, when drying, silicone is too hard for an adequately dissection. Epoxy resin advantages are its low viscosity that allows small vessels perfusion and, once epoxy resins catalyzed, it does not collapse. Its disadvantages include its toxicity (epoxy resins have been related to cancer development) and, when catalyzed, it turns so hard that makes dissection more difficult (37). Finally, latex advantages include its low viscosity, excellent perfusion of small vessels and, because latex does not catalyze, it stays soft that is very helpful for vascular dissection. Disadvantages of latex are that it does not catalyze, so it does not get hard and, although latex maintains perfused and color, vessels collapse (38). The increase in tissue stiffness after formaldehyde fixation is both an advantage and a limitation depending on the intended use of the specimen. For anatomical studies and surgical training, the increased stiffness can help preserve the specimen's structural details, allowing for repeated handling and examination without significant degradation. This is crucial in neurosurgical training, where understanding the intricate anatomy of the brain and its vascular system is essential for developing surgical skills. However, the increased stiffness can also introduce a limitation by reducing the realism of the surgical simulation. In a live surgical scenario, tissues exhibit a certain degree of flexibility and elasticity, which are important for the surgeon to experience and learn how to navigate during delicate procedures. The stiffness induced by formaldehyde fixation can make the tissue less realistic in terms of texture and response to surgical manipulation, potentially affecting the training experience. Furthermore, cadaver-based training offers a safe and ethical platform for practicing surgical procedures. Trainees can perform repeated dissections and surgical simulations without the risk of harm to living patients. This aspect of cadaveric learning is particularly important in neurosurgery, where precision and accuracy are paramount, and the margin for error is minimal. The cadaveric method also allows for the practice of managing complications, providing an invaluable learning experience that is difficult to replicate in other training environments (39).

### Vascular dissection and preservation in neurosurgical training

**Figure 7:**
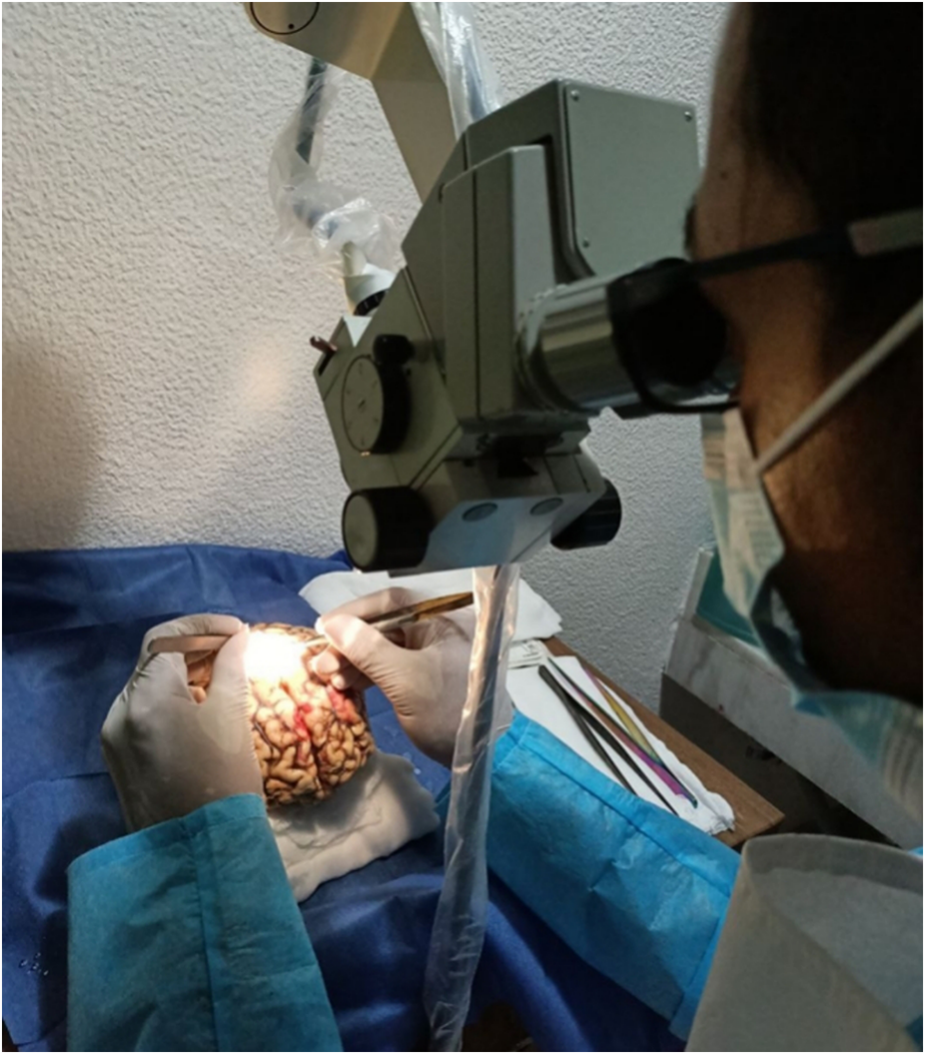
A surgeon during training.

In neurosurgical training, a pivotal skill set involves the strategic dissection around major blood vessels and preserving vascular integrity, particularly in surgeries involving the brain's surface or deep-seated structures (40). Training focuses on safely navigating and dissecting around major blood vessels. This includes learning to identify and isolate vessels, understanding the layers of brain tissue, and the use of microdissection tools to gently separate vessels from the surrounding neural structures (41). Preserving the integrity of vessels during dissection is crucial. Techniques taught include handling methods that minimize traction and prevent vessel tearing or puncture (Figure 7). This skill is particularly vital in surgeries involving vascular structures near sensitive areas of the brain (40).

### Application in Various neurosurgical procedures

Residents learn to dissect around the vasculature in various scenarios, such as brain tumor resection and intracranial hemorrhage management. The training includes simulations of complex cases where vascular preservation is key, such as operations involving the brain's eloquent areas or deep-seated structures (42). Critical decision-making skills are honed, focusing on when to preserve or sacrifice vessels based on surgical priorities and patient safety (43).

#### Microvascular decompression training

Training includes understanding the pathophysiology behind conditions like trigeminal neuralgia or hemifacial spasm, often caused by vascular compression of cranial nerves. Residents learn specific techniques for microvascular decompression (MVD), including how to identify offending vessels and safely mobilize them away from the nerve (44, 45). Using latex-injected models, residents can simulate MVD procedures, practicing the delicate balance between relieving nerve compression and preserving vascular integrity. The training can lead to emphasize the use of fine microsurgical instruments to manipulate both the nerve and the vessel, fostering dexterity and precision.

#### Skull base surgery simulation

Training with latex-injected models plays a pivotal role in integrating advanced surgical approaches, particularly in the realms of skull base surgery and the development of endoscopic skills. These models provide a realistic and detailed platform for residents to practice and refine their techniques in these specialized areas (42). The latex-injected models enable residents to simulate various skull base approaches. These include techniques to access the anterior, middle, and posterior cranial fossae, each presenting unique challenges and requiring precise surgical maneuvers. Such training is crucial for understanding the complex anatomy of the skull base, including its neurovascular structures (46, 47). The models allow for the simulation of realistic practice of complex procedures like pituitary tumor resections, acoustic neuroma removal, and the management of skull base meningiomas (48). Through these simulations, residents gain a deeper understanding of the spatial relationships and surgical anatomy involved in these areas, which are often difficult to access and require meticulous dissection (49). A key aspect of skull base surgery training is refining skills like bone drilling, dural opening, and the delicate manipulation of brain tissue, all essential for minimizing patient morbidity. The tactile feedback from the models is invaluable in understanding the nuances of working within the confined spaces of the skull base.

#### Endoscopic skills

The adoption of endoscopic techniques in neurosurgery has significantly enhanced the minimally invasive management of various brain and spinal conditions. Training includes the use of endoscopes for procedures like endoscopic third ventriculostomy, pituitary surgeries, and ventricular tumor biopsies (50). An essential part of endoscopic training is developing hand-eye coordination, adapting to the unique visualization provided by endoscopes. Unlike traditional microscopic surgery, endoscopic views require a distinct set of motor skills and spatial awareness (49, 50). Residents practice a range of endoscopic approaches, including transnasal and transsphenoidal routes to the skull base, as well as intraventricular pathways. This training is vital for learning to navigate through the narrow anatomical corridors and manipulating instruments within the limited visual and spatial confines inherent in endoscopic procedures (51). These exercises challenge residents to select the most suitable surgical approaches and techniques for different scenarios. This process is critical and involves a comprehensive evaluation of patient-specific factors, pathology characteristics and potential surgical outcomes (52). The training often involves collaborative problem-solving, where residents engage in group discussions, fostering a team-based approach to surgical planning (53).

#### Complex surgical procedures

As residents advance in their training, they are introduced to more complex neuro-surgical procedures. This includes intricate resections of deep-seated tumors, and the management of complex aneurysms, requiring a high level of surgical skill and expertise (54–57). Repeated practice on the latex-injected models is essential for refining surgical techniques. This practice enables residents to develop a nuanced understanding of surgical maneuvers, enhancing their precision and dexterity. An integral part of the neurosurgery training is continuous feedback from mentors and self-evaluation. Residents are encouraged to critically assess their techniques, learn from their experiences, and make steady improvements.

### Continuous assessment, feedback and research in neurosurgical training

A key component of neurosurgical training involves the comprehensive skill assessment of residents. This assessment covers a broad range of competencies essential to the practice of neurosurgery. Instructors evaluate technical skills such as the precision and accuracy of surgical techniques, decision-making abilities under stressful and complex situations, and proficiency in the handling and manipulation of various neurosurgical instruments. But the assessment goes beyond just technical prowess (58, 59). Feedback provided to residents is objective, structured, and meticulously focuses on specific aspects of their performance. It encompasses areas beyond technical skills, including patient care, professionalism, ethical practice, and interpersonal and communication skills. This holistic approach ensures the well-rounded development of future neurosurgeons (52, 55). During surgical simulations and actual procedures, instructors closely monitor the residents’ performance, providing immediate and constructive feedback. This real-time evaluation is crucial as it allows residents to understand and correct their techniques and decisions promptly, reinforcing learning and improvement in the moment. The ethos of neurosurgical training heavily emphasizes the importance of repetitive practice. Residents are encouraged to engage in various surgical scenarios repeatedly. This consistent practice allows them to continually hone their surgical techniques, improve decision-making, and solidify their understanding of complex neurosurgical procedures (60). A crucial aspect of this training is the opportunity to gain experience from mistakes in a controlled and supportive environment. This learning process is invaluable as it builds resilience, adaptability, and critical analytical skills, qualities that are indispensable for neurosurgeons. Iterative learning in neurosurgical training is progressive. As residents advance in their skills and knowledge, the complexity of the training scenarios also increases. This progression challenges residents to continuously enhance their skills and adapt to more demanding surgical situations.

In the evolving landscape of neurosurgery, the integration of clinical training with research and academic endeavors plays a pivotal role. Latex-injected brain models, can become a cornerstone in neurosurgical training, provide residents with a unique opportunity to contribute to the advancement of the field through innovative research and academic pursuits. In addition to practical skills, working with cadavers helps in cultivating a sense of respect and empathy among medical trainees towards their future patients. The experience often instills a sense of the gravity and responsibility inherent in surgical roles, reinforcing the ethical dimensions of medical practice, cadaver-based training is also essential for advancing surgical techniques and innovations (55). It provides a platform for experienced surgeons to refine new procedures and for researchers to explore and understand complex anatomical relationships. Many groundbreaking surgical techniques and instruments have been developed and perfected through cadaveric studies. However, the efficiency of the cadaveric method is not without its challenges. The availability of cadavers is limited, and there are significant costs and ethical considerations involved in their procurement and use. Moreover, the preservation techniques, such as embalming, can alter tissue properties, which may affect the realism of the training experience (35).

### Anatomical specimens and novel surgical simulators

In the evolving landscape of surgical training, the juxtaposition of traditional anatomical specimens with new surgical simulators, such as UpSurgeon and augmented reality head-mounted displays, outlines a fundamental shift in how surgical skills are developed and refined (61–64). Each educational tool brings its own advantages and limitations to the forefront, shaping the contours of medical education in distinct ways. Traditional anatomical specimens have long been the cornerstone of medical and surgical education, prized for their unparalleled realism. The tactile feedback, intricate structural nuances of tissues, and the complexity of anatomical variations they offer are invaluable (64, 65). They afford medical students and surgical residents an authentic experience of human anatomy, allowing for a comprehensive exploration of the complex relationships between various structures within a true three-dimensional space. However, the use of cadaveric specimens is not without its challenges. Ethical considerations, availability and the logistical and financial burdens associated with their maintenance pose significant hurdles. Additionally, while cadavers offer an accurate representation of human anatomy, they often lack the diversity of pathological conditions surgeons will face in actual practice. Another notable limitation is their one-time use: once a dissection or surgical procedure has been performed, the opportunity for repeated practice on the same anatomy is lost. On the other side of the spectrum, novel surgical simulators like UpSurgeon introduce a paradigm of accessibility and repeatability to surgical training and are less expensive than real heads (62, 66–68). These virtual platforms allow students and young surgeons to engage in surgical procedures multiple times, free from the logistical constraints tied to cadaveric specimens (67, 68) and offer a wide array of pathological conditions, providing learners with exposure to various clinical scenarios. Moreover, many of these simulators offer immediate feedback on performance, encompassing aspects such as precision, timing and decision-making (13, 14, 69). This feature is invaluable for self-assessment and progressive skill improvement. However, these advanced simulators have some limitations, as the effectiveness of simulation-based learning is also contingent on the learner's ability to adapt to the virtual or augmented reality environment. A hybrid educational strategy that synergizes the strengths of both traditional anatomical specimens and novel surgical simulators could represent the most comprehensive approach to surgical training (63, 64). Such an integrated approach would not only ensure a breadth of knowledge and skill but also cultivate adaptability and proficiency in future surgeons, preparing them effectively for the dynamic and evolving demands of surgical practice.

### Limitation of the study

This study has some limitations. The natural specimen variability in human anatomy can introduce inconsistencies in the results. Factors like age, sex and individual anatomical differences can affect the outcomes of the injection and dissection processes. The use of formaldehyde and other preservation methods can alter the tissue properties, potentially affecting the realism of the training experience. The hardness of preserved tissues may not accurately mimic the conditions of live surgery. The extraction process, despite being meticulously conducted, carries a risk of damaging the cerebral vascular system, which can impact the subsequent steps of injection and dissection. The manual washing of the vascular system and the perfusion processes are delicate and can result in the rupture of smaller vessels if not done with extreme care. While latex offers several advantages, it does not harden like other materials, leading to potential collapse of the vessels after injection. This can limit the realism in mimicking blood flow dynamics during surgical training. There is always a risk of leakage during the latex injection process, and in some cases, incomplete perfusion may occur due to anatomical variations or existing vascular pathologies.

## Conclusions

Latex vascular injection technique of the brain and head-neck segment make possible the development of simulators for neurosurgical training with a real experience that could improve surgical skills and results. Authors recommend latex injection for neurosurgical training because its smoothness makes possible a good dissection; although silicone provides a better visual effect of the vessels, is more helpful for illustrations than for training.

## Data Availability

The raw data supporting the conclusions of this article will be made available by the authors, without undue reservation.
